# Continous surveillance of bloodstream infections across departments at a Danish University Hospital

**DOI:** 10.1186/1753-6561-5-S6-P64

**Published:** 2011-06-29

**Authors:** RA Leth

**Affiliations:** 1 Department of Clinical Micorbiology, Aarhus University Hospital, Skejby, Aarhus N, Denmark

## Introduction / objectives

The rate of bloodstream infections has been monitored monthly since 2004 at Aarhus University Hospital, Skejby. The hospital is a tertiary referral hospital with 400 beds specialising in paediatrics, gynaecology & obstetrics, urology, thoracic and vascular surgery, renal medicine, cardiology, infectious diseases and intensive care.

The aim of this study was to identify variations in bloodstream infection rates across departments.

## Methods

Monthly all patients with positive blood cultures are monitored concerning true infection or contamination and if the infection is community-or hospital/healthcare-acquired. The bloodstream infection rates are presented as number of episodes per 1000 beddays. The infection rates are sent to each department quarterly followed by staff-meetings. To identify trends in bloodstream infection rates we also perform six-month moving averages.

## Results

In the years 2007 - 2010 the total bloodstream infection rates for the hospital were 3.0, 3.1, 2.9, and 2.6 episodes per 1000 beddays. Hospital-acquired infections accounted for 2.2, 2.0, 2.0, and 2.0 episodes per 1000 bed-days. There were great differences in hospital-acquired bloodstream infection rates across departments ranging from 0.5 episodes per 1000 beddays in gynaecology & obstetrics to 5.3 episodes per 1000 beddays in urology in 2010.

## Conclusion

The overall rate of hospital-acquired bloodstream infections has remained at the same level in the past four years at our hospital. However, some departments have a high rate constituting a continuous challenge for improvement.

## Disclosure of interest

None declared**.**

